# Postpartum Hypocupric Myelopathy Masquerading as Acute Transverse Myelitis: A Case Report and Literature Review of a Rare Presentation

**DOI:** 10.7759/cureus.52149

**Published:** 2024-01-12

**Authors:** Nikhil Pantbalekundri, Sunil Kumar, Sourya Acharya, Gautam Bedi

**Affiliations:** 1 Department of General Medicine, Jawaharlal Nehru Medical College, Datta Meghe Institute of Higher Education and Research, Wardha, IND

**Keywords:** hyperzincemia, hypocupremia, copper-induced myelopathy, copper deficiency, acute transverse myelitis (atm)

## Abstract

The symptoms of transverse myelitis, an acute demyelinating inflammatory condition of the spinal cord, include motor, sensory, and bowel-bladder dysfunction that can develop suddenly or gradually. Several etiologies, such as bacterial, fungal, or viral infections, cancer, autoimmune diseases, vascular problems, and environmental variables, can cause it. The identification of copper deficiency myelopathy (CDM) as a curable cause of non-compressive inflammatory myelopathy has only occurred recently. Patients frequently present with sensory complaints and a spastic gait. The neurological disease may exist independently of the hematologic signs. Only a few cases of copper myelopathy in peripartum women have been documented. Given that hypocupric myelopathy is a treatable cause of debilitating paraplegia, maintaining clinical vigilance will be crucial in minimizing neurological sequelae, as demonstrated in this case report.

## Introduction

Acute transverse myelitis, an acquired spinal cord disorder, usually presents with rapid-onset weakness, sensory deficits, and autonomic dysfunction. Transverse myelitis can result from several established causes, including bacterial and viral infections, vaccinations, and vascular conditions such as anterior spinal artery thrombosis, vasculitis, spinal arteriovenous malformation, and paraneoplastic syndromes. Several hypothesized etiologies suggest that myelin proteins are inadvertently impacted by the immune system's reaction to a foreign infection. Since acute transverse myelitis is associated with autoimmunity, inflammatory, and viral illnesses, determining the etiology of transverse myelitis can be difficult.

Many metalloenzymes and proteins that preserve the nervous system's structure and function require copper. Dietary deficits are rare due to their wide meal dispersion and low daily demand. Hypocuprism can manifest as myelo-neuropathy and mimic subacute combined degeneration (SACD) or transverse myelitis. It manifests as a progressive condition with clinical, electrophysiological, and radiographic signs of myelopathy [1]. The precise cause of copper deficiency is unknown. However, factors that are known to contribute include nephrotic syndrome, excessive zinc therapy, upper gastrointestinal surgeries, enteral or parenteral feeding without copper supplements, and starvation. Moreover, demyelination is thought to be caused by a flaw in luminal copper transport or trafficking [2,3]. There is a paucity of research on hypocuprism-related copper myeloneuropathy, and there are few reported cases of copper myelopathy during the peripartum period. Recognizing the neurological aftereffects of transverse myelitis is crucial since patients respond effectively to treatment.

## Case presentation

A 38-year-old female presented with a 15-day history of rapidly progressive ascending weakness and stiffness in both her lower limbs. It was associated with numbness, tingling, and an inability to control urination and bowel movements. This weakness in bilateral lower limbs was insidious in onset and rapidly progressive over 15 days, such that the patient required support to mobilize, restricting her movements to her bed. It was associated with tingling, numbness, and an inability to sense water temperature while bathing. Further, the patient also complained of constipation and an inability to control the urge to urinate, for which the patient was catheterized. The patient was six months postpartum with an uneventful antenatal period followed by a full-term normal vaginal delivery of a healthy male child. She gave a drug history suggestive of intake of various multivitamins over one year as a part of her antenatal care after consulting multiple doctors for her pregnancy. She had her routine vaccination done during her antenatal period. There is no history of girdle-like sensations, any recent infections, or similar complaints in the past. The patient consumed a mixed diet and gave no history of previous hospitalization.

Neurological examination revealed clasp knife rigidity, hyperreflexia in the lower limbs compared to the upper limbs, and significantly decreased power in both lower limbs (grade 3/5). Among superficial reflexes, the abdominal reflex was preserved; while both plantar reflexes were extensors, the anal wink reflex and inguinal reflex were absent. The vibration and joint position senses were impaired bilaterally up to the iliac spine. Fine touch, temperature, and pressure sensations were lost up to thigh bilaterally. She had a spastic ataxic gait on further examination.

Her blood investigations were as mentioned (Table 1). Serum electrolytes, serum iron studies, serum vitamin B12 levels, and thyroid profile were within the normal range. The serology for human immunodeficiency virus (HIV) was negative. With all the usual causes ruled out, additional investigations were done. These included anti-nuclear antibodies (ANA), anti-phospholipid antibodies IgG, and IgM, as well as assessments of serum copper, 24-hour urinary copper, serum ceruloplasmin, and serum zinc.

**Table 1:** Investigations during the hospital stay. TSH, thyroid stimulating hormone

Investigations	Value	Normal range
Complete blood count		
Hemoglobin	9.6 g/dL	13-17 g/dL
White blood cells	7,800/cmm	4,500-10,500/cmm
Platelets	380,000/cmm	150,000-400,000/cmm
Kidney function test		
Serum urea	36 mg/dL	6-24 mg/dL
Serum creatinine	1.3 mg/dL	0.7-1.3 mg/dL
Liver function test		
Alkaline phosphatase	151 U/L	90-300 U/L
Serum glutamate pyruvate transaminase	36 U/L	0-45 U/L
Serum glutamate oxaloacetate transaminase	49 U/L	0-50 U/L
Albumin	4.0 gm/dL	3.5-5 gm/dL
Total protein	6.7 gm/dL	6-8.5 gm/dL
Total bilirubin	1.1 mg/dL	0-1 mg/dL
Conjugated bilirubin	0.4 mg/dL	0-0.35 mg/dL
Unconjugated bilirubin	0.7 mg/dL	0-0.65 mg/dL
Other investigations		
Serum vitamin B12	1,000 µg/dL	240-950 µg/dL
Serum iron	58 µg/dL	60-160 µg/mL
Serum ferritin	12 ng/mL	6.24-137 ng/mL (for female less than 50 years old)
Serum free T3	5 pg/mL	2.77-5.27 pg/mL
Serum free T4	1.3 ng/dL	0.78-2.19 ng/dL
Serum TSH	4.2 µIU/mL	0.465-4.68 µIU/mL
Serum copper	48 µg/dL	80-155 µg/dL (female)
Serum ceruloplasmin	60 mg/dL	20-40 mg/dL
Serum zinc	168 µg/dL	60-150 µg/dL
Urinary copper	28 µg/24 hour	20-50 µg/24 hour
Anti-nuclear antibodies (ANAs) by immunofluorescence technique	0.8	Negative if less than 0.9
Anti-phospholipid antibody (IgG)	3.8 AU/mL	<10 AU/mL
Anti-phospholipid antibody (IgM)	2.7 AU/mL	<10 AU/mL

**Figure 1:**
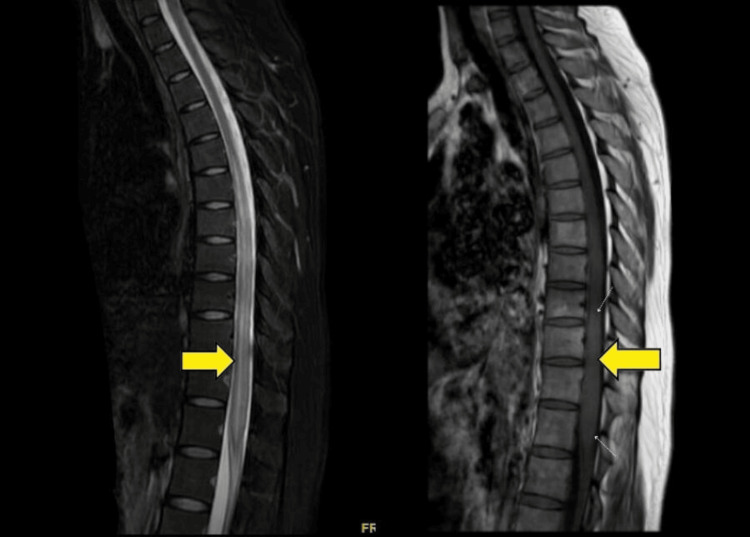
Contrast-enhanced MRI of the dorsolumbar spine: (A) MRI T2 images suggestive of the intramedullary hyperintense lesion at the D7-D9 level (yellow arrow); (B) MRI T1 image showing a long-segment hypointense lesion at the D7-D9 level (yellow arrow). MRI, magnetic resonance imaging

With suspicion of non-compressive myelopathy at an upper lumbar spinal segmental level based on clinical examination, a magnetic resonance imaging (MRI) of the dorso-lumbar and lumbosacral spine was ordered. It indicated intramedullary, long-segment T2 hyperintensity in the spinal cord, extending from the lower endplate of the D7 vertebra to the upper endplate of the L1 vertebra, with patchy enhancement observed in the D8-D9 segment on the post-contrast image (Figure 1), suggestive of myelitis features.

Multivitamins were stopped. Oral supplementation of elemental copper equal to 1,500 µg/day was provided. The patient was started on intravenous methylprednisolone 1 gm once a day for five days, following which the patient was switched to oral prednisolone 40 mg once a day, which was continued for 30 days. After five days of intravenous corticosteroid therapy, the patient had significant improvement in her symptoms. The weakness recovered over six days of hospital stay, by the end of which the patient could stand on her own and walk with support. The frequency of involuntary evacuation of bladder and bowel movements decreased. However, impaired vibration and joint sense persisted up to the ankle joint, and gait was spastic. The patient was simultaneously started on lower limb physiotherapy for the same. After two months of follow-up, the patient reported functional improvement and an enhanced quality of life.

## Discussion

Transverse myelitis, a rapidly evolving disease, is caused by affection of the spinal cord in response to inflammation or infection. With an annual incidence of up to 3 per 100,000 patient-years (0.003%), it is a relatively uncommon illness, with a higher frequency in females [4]. When a thorough workup fails to identify an underlying cause, a case is classified as idiopathic transverse myelitis. Secondary causes of disease-related transverse myelitis include infections, connective tissue disorders, vitamin B12 deficiency, recent vaccinations, and multiple sclerosis [5]. While hematological features are the primary presentation of copper deficiency, copper deficiency myelopathy (CDM) is a typical presentation in all cases of neurological manifestations of copper insufficiency [6].

A daily recommended dietary allowance of 0.9 mg for copper makes it a necessary dietary micronutrient [7]. Most copper absorption happens in the duodenum. After copper is absorbed through the apical membrane transporter Ctr1, whose expression is controlled by copper status, P-type ATPase (ATP7A), lacking in Menkes' disease, transfers copper across the basolateral membrane. Iron and zinc can reduce the absorption of intestinal copper [8]. Copper is bound to albumin in the portal circulation. Copper status can be determined by measuring serum ceruloplasmin. In redox processes, copper predominantly functions as an electron transfer mediator in hepatocytes. It is a catalytic component of several essential enzymes, primarily monooxygenases and oxidoreductases. With the help of a P-type ATPase (ATP7B) absent in Wilson's illness, copper is released into the bile. The primary homeostasis mechanism is biliary excretion, and chronic copper poisoning is unlikely to occur in the absence of an acquired (hepatic failure) or hereditary (Wilson's disease) secretory deficit [9]. While most of the released copper is reabsorbed, just a small quantity (30-60 μg/day) of copper enters the urine, and 0.5-1.5 mg/day ends up in the feces. The chelator metallothionein is expressed more frequently in enterocytes when zinc and iron are present. Compared to zinc, copper has a stronger affinity for metallothionein and stays bound in the enterocytes until being shed into the lumen and excreted. Hyperzincemia in his study's patient may have resulted from a prolonged intake of a multivitamin supplement containing zinc. The primary cause of malabsorption in CDM is celiac disease [10]. Specific micronutrients may have a smaller effective absorption area following certain forms of upper gastrointestinal surgery. Since its initial description in 2001, the correlation between a remote history of upper gastrointestinal surgery, copper deficiency, and myelopathy has been documented more frequently after undergoing bariatric and non-bariatric gastrointestinal interventions [11]. A few documented causes of copper metabolism are mentioned in Table 2.

**Table 2:** Causes of copper deficiency.

Causes of copper deficiency
Dietary deficiency
Menkes disease
Enteropathies such as inflammatory bowel disease and celiac disease
Zinc supplement overuse, parenteral overdosing, or denture cream ingestion
Iron supplement overuse
Excessive use of copper chelators
Small intestinal bacterial overgrowth
Upper gastrointestinal surgery: bariatric and non-bariatric upper gastrointestinal interventions
Prolonged intravenous nutrition (total parental nutrition)

The most common hematological signs of copper deficiency are neutropenia or anemia. The most commonly described neurological symptom of copper deficiency is myelopathy [12]. The hypothesized cause of CDM is the malfunction of three copper-dependent enzymes: S-adenosylhomocysteine hydrolase (which catalyzes the final movement of a methyl group from methyl-tetrahydrofolate to several kinds of macromolecules, including myelin proteins), cytochrome-c oxidase (which prevents lactate formation), and methionine synthase [13]. These enzymes ultimately cause the demyelination of nerves. A poorly defined, hyperintense lesion in the spinal cord that varies in size and extends up to three or four spinal segments can be seen on T2 images of a contrast-enhanced MRI of the spine, which is the primary method used to diagnose CDM [14].

The cornerstone of treatment was copper supplementation, which was usually administered intravenously at first [15,16] or orally at first [17]. It is recommended to take doses comparable to 1 to 2 mg/day of elemental copper [18]. Copper acetate, copper chloride, copper citrate, copper gluconate, copper histidine, and copper sulfate were among the copper salts employed. Treatment for the underlying cause of hypocupremia involves avoiding any external source of zinc or iron, adhering to a gluten-free diet for celiac disease, undergoing gastric bypass surgery, using steroids for mesangio-proliferative glomerulonephritis, and using antibiotics to address small intestinal bacterial overgrowth [19]. According to a large body of research on curable causes of transverse myelitis, CDM is frequently detectable early and critical for patient prognosis [20].

## Conclusions

CDM is an under-recognized syndrome causing non-compressive myelopathy. Serum copper levels should be estimated as part of the workup for patients with myelopathy or myeloneuropathy, especially if there are factors for copper deficiency. Anemia and neutropenia are frequent hematologic symptoms but are not always present. While further investigation into the pathophysiology of CDM is required, the neurological prognosis and quality of life of patients affected by myelopathy can be significantly improved by early identification and treatment of curable causes.
